# Editorial: Macrophage immunity and metabolism in cancer: Novel diagnostic and therapeutic strategies

**DOI:** 10.3389/fimmu.2022.1113031

**Published:** 2022-12-16

**Authors:** Jianmei W. Leavenworth, Xin Lai, Hongming Miao, Di Wang, Huakan Zhao, Yongsheng Li

**Affiliations:** ^1^Department of Neurosurgery, Heersink School of Medicine, University of Alabama at Birmingham, Birmingham, AL, United States; ^2^The O’Neal Comprehensive Cancer Center, University of Alabama at Birmingham, Birmingham, AL, United States; ^3^Department of Microbiology, Heersink School of Medicine, University of Alabama at Birmingham, Birmingham, AL, United States; ^4^Laboratory of Systems Tumor Immunology, Department of Dermatology, Friedrich-Alexander-Universität Erlangen-Nürnberg (FAU) and Universitätsklinikum Erlangen, Erlangen, Germany; ^5^BioMediTech, Faculty of Medicine and Health Technology, Tampere University, Tampere, Finland; ^6^Department of Biochemistry and Molecular Biology, Third Military Medical University (Army Medical University), Chongqing, China; ^7^Department of Biochemistry and Molecular Biology, Southwest Medical University, Luzhou, China; ^8^Institute of Immunology and Sir Run Run Shaw Hospital, Zhejiang University School of Medicine, Hangzhou, China; ^9^Department of Medical Oncology, Chongqing University Cancer Hospital, Chongqing, China

**Keywords:** macrophage, tumor-associated macrophages, tumor microenvironment, tumor immunity, cancer immunotherapy

The immunosuppressive tumor microenvironment (TME) represents one of major hurdles for cancer immunotherapy. To reprogram the immunosuppressive cold TME into an immune active hot TME, many of current immunotherapeutic strategies have focused on reviving exhausted T cells and also targeting various immunosuppressive cells, particularly tumor-associated macrophages (TAMs) (1). Thus, a better understanding of the functional plasticity of TAMs and the factors responsible for modulating their function would facilitate to develop the most effective TAM-based therapeutic strategies. These subjects are presented and discussed in a collection of articles in this Research Topic.

The immune landscape within each type of tumor is often distinct. Lv et al. provided a comprehensive analysis of 22 different types of tumor-infiltrating immune cells in the uveal melanoma (UM) microenvironment by leveraging the CIBERSORT, a deconvolution methodology, which decoded tumor-infiltrating immune cell (TIC) profiles from the bulk RNA sequencing datasets extracted from the public resources (TCGA and GEO). This approach combined with unsupervised clustering methods and other computational algorithms was allowed to develop the TIC score as a prognostic biomarker and immune therapeutic predictor for UM patients. Specifically, patients with a better prognosis and favorable response to immune checkpoint therapy expressed the high-TIC score signature, which was reflected by an upregulation of genes related to immune checkpoints (e.g., PD-1, CTLA-4) and immunological activity, including those enriched for proinflammatory M1 macrophages. This analysis pipeline could be utilized to characterize the immune landscape and discover biomarkers in other types of cancer.

With the technology advancement, profiling the TME can be performed at the single-cell resolution combined with multi-omics analysis (2, 3). Yang et al. adopted the single-cell RNA sequencing, whole-exome sequencing and microbiome sequencing to compare the tumor immune signatures of synchronous primary colorectal cancers to those of gastric cancers (GC). Despite that the two types of cancer shared many similarities reflected by a similar cellular distribution pattern within the juxta-tumoral normal tissues in both cancer samples, their TME was quite distinct with a marked increase of macrophages and myeloid cells in the GC tumoral sites. Interestingly, this study also revealed that the cellular components in the TME were influenced by the tumor mutational landscape and metabolism-related microbiome, providing insights for future investigation of these types of cancer.

TAMs are actually heterogenous comprising different subsets with distinct capacity to modulate tumor immunity (4). Among the various subsets, M1-like cells are pro-inflammatory and exhibit anti-tumor properties, while M2-like cells are often pro-tumoral and immunosuppressive. Thus, eliminating M2-like TAMs may offer therapeutic potential, but which often lacks the specificity resulting in wide-spread side effects. Instead, repolarizing pro-tumoral TAMs towards the pro-inflammatory M1-like phenotype is suggestive of a better treatment option. This strategy was evidenced by Vadevoo et al. The authors demonstrated that a combined use of the DNA methylation inhibitor 5-aza-2’-deoxycytidine and the histone deacetylation inhibitor trichostatin A decreased M2-type macrophages while increasing M1-type and improved anti-tumor immunity, leading to an efficient tumor control. Mechanistically, the combination treatment upregulated miR-7083-5p in M2 macrophages, which curtailed tumor growth, in part by inhibiting the expression of several genes involved in tumor progression. These preclinical results suggested that reprogramming immune cells, including TAMs, *via* epigenetic therapy may be a promising approach to treat cancer (5).

Lastly, Li et al. summarized three types of innate T cells that can reprogram the TME by targeting both tumor and immunosuppressive cells, particularly TAMs and myeloid derived suppressor cells. These innate T cells, including invariant natural killer T cells, mucosal-associated invariant T cells and gamma delta T cells, are great candidates for cell-based immunotherapy and can be engineered *via* the chimeric antigen receptor-based technology. One of the therapeutic advantages of these innate T cells over conventional T cells for the allogeneic engraftment is that these cells do not require MHC antigen presentation for recognition and function, minimizing the risk of graft versus host disease. This review also discussed the specific mechanisms by each of these innate T cells adopt to disrupt TAM-mediated immunosuppression while preserving the pro-inflammatory function of activated macrophages, and pointed out the limitations and improvement strategies for their use in cell-based therapies for the treatment of solid tumors.

## Conclusions

Our Research Topic “*Macrophage Immunity and Metabolism in Cancer: Novel Diagnostic and Therapeutic Strategies*” has stimulated the discussion of current research on the immunosuppressive TME, the macrophage-mediated regulation of tumor immunity and new macrophage-based strategies for cancer treatment. We (the editors) believe that this Research Topic has provided a platform for future studies of macrophage immunity and macrophage-based cancer immunotherapies.

## Author contributions

JWL drafted this editorial article. All authors made substantial, direct and intellectual contributions to the work, and approved it for publication.
